# OGG1 and MUTYH DNA Glycosylases, the Dynamic Duo Against 8-Oxoguanine DNA Lesion: Structure, Regulation, and Novel Emerging Roles

**DOI:** 10.3390/biom16020257

**Published:** 2026-02-05

**Authors:** Ana P. Gómez-Ramírez, Melody Malek, Estela G. García-González, Sergio E. Campos, Luis G. Brieba, Sheila S. David, Carlos H. Trasviña-Arenas

**Affiliations:** 1 Centro de Investigación Sobre el Envejecimiento, Centro de Investigación y de Estudios Avanzados (CINVESTAV), Unidad Sede Sur, Tlalpan, Ciudad de México 14330, Mexico; ana.gomez.ramirez@cinvestav.mx (A.P.G.-R.); estela.garcia@cinvestav.mx (E.G.G.-G.); sergio.e.campos@cinvestav.mx (S.E.C.); 2 Chemical Biology Graduate Program, Department of Chemistry and Chemistry, University of California, Davis, CA 95616, USA; mmalek@ucdavis.edu (M.M.); ssdavid@ucdavis.edu (S.S.D.); 3 Laboratorio Nacional de Genómica para la Biodiversidad, Centro de Investigación y de Estudios Avanzados (CINVESTAV), Irapuato 36821, Mexico; luis.brieba@cinvestav.mx

**Keywords:** oxidative DNA damage, DNA glycosylase, MUTYH, OGG1, DNA repair

## Abstract

OGG1 and MUTYH are base excision repair (BER) DNA glycosylases (DGs) from the Helix–hairpin–Helix superfamily responsible for initiating and coordinating the repair of 8-oxo-7,8-dihydroguanine (OG), and its replication-derived mispair with adenine (OG:A), respectively. The DNA repair activities of these DGs are pivotal to safeguarding nuclear and mitochondrial genomes. Indeed, DG functional impairment is associated with numerous pathologies, including neurodegenerative diseases, metabolic syndromes, and cancer. The timely and precise localization and processing of oxidized nucleobases carried out by these DGs are modulated by a complex regulatory network at both transcriptional and posttranslational levels, as well as intricate protein–protein interaction networks. In the absence of regulation, inappropriate and imbalanced DG activity may trigger telomeric instability, changes in transcriptional profiles and cell death. This review focuses on summarizing key features of OGG1 and MUTYH function, with a special emphasis on structure, regulation, and novel emerging roles.

## 1. Introduction

**Figure 1:**
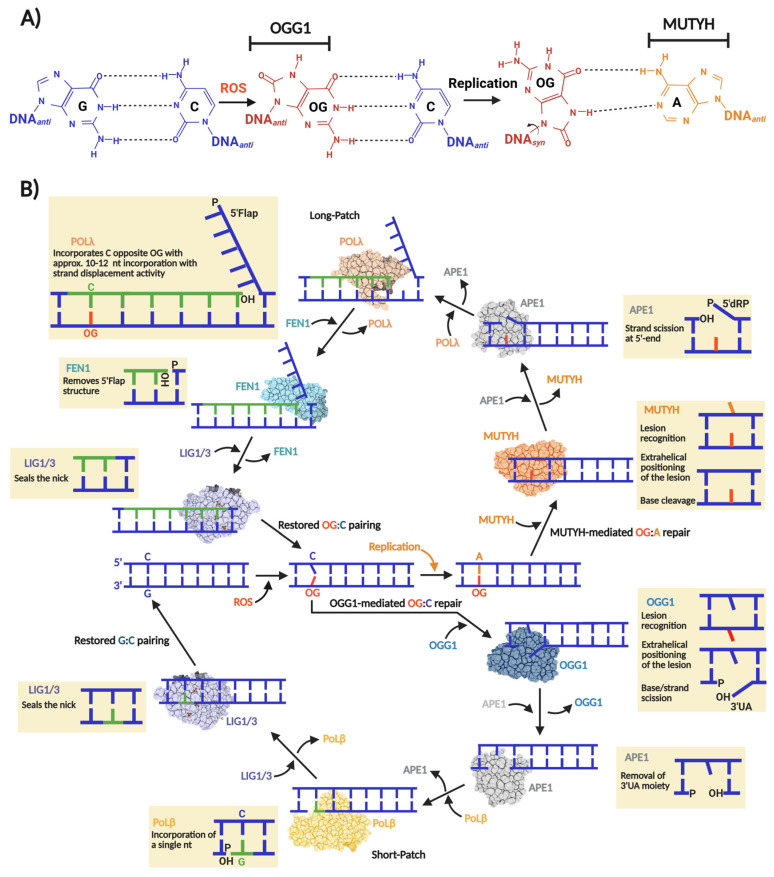
Base excision repair of OG:C and OG:A in human cells. (**A**) OG miscoding potential arises from anti versus syn nucleotide conformations that allow for distinct base pairing patterns. The syn conformation of OG presents the Hoogsteen face, instead of the canonical Watson–Crick face, favoring the incorporation of an adenine opposite OG during a replication event initiating a GC→TA transversion. The nucleobases subjected to OGG1 and MUYTH repair are indicated. (**B**) “One-reaction–one enzyme” scheme representing the flow of enzymatic reactions to repair OG:C and OG:A via OGG1- and MUTYH-mediated BER short- and long-patch, respectively. In the case of OG:C repair, OGG1, as bifunctional DG, carries out OG excision producing an AP site that thereafter is converted to 3′-α,β-unsaturated aldehyde (3′UA) via β-elimination. The 3′UA is processed by APE1 leaving a 3′-OH nick which is used by Polβ and a DNA ligase (LIG1 or LIG3) to incorporate a G and ligate the nick, respectively. In the case of OG:A repair, MUTYH, as monofunctional DG, carries out only the adenine excision opposite OG, producing an AP site which in turn is processed by APE1 leaving a 5′-2-deoxyribose-5-phosphate (5′dRP) and a 3′-OH nick. Then, Polλ incorporate several nucleotides (10–12) generating a 5′Flap structure. To consolidate the repair, the 5′Flap is processed by FEN1 and the remaining nick ligated by LIG1 or LIG3.

A hallmark of oxidative DNA damage is the oxidation of guanine to 8-oxo-7,8-dihydroguanine (OG), which forms and distributes unevenly within our genome, with basal levels estimated around 10,000 OG per mammalian cell [1,2]. The highly oxidizing environment of mitochondria results in a 10-fold higher accumulation of OG than in the nucleus. The molecular issue with OG pertains to its highly mutagenic nature, which is underscored by the fact that DNA polymerases tend to incorporate an adenine (A) opposite OG, inducing G:C→T:A transversion mutations [3] (Figure 1A).

The replication-associated miscoding of OG is problematic across organisms in all domains of life. Consequently, a highly conserved enzymatic pathway in archaea, bacteria, and eukaryotes mitigates OG-induced mutagenesis. This pathway, first described by Michaels and Miller as the “Guanine Oxidation” (GO) repair system, consists of three enzymes: MutM, MutY, and MutT [4,5]. MutM, also known as formamidopyrimidine glycosylase (Fpg), is a Helix–2Turn–Helix (H2TH) DNA glycosylase found primarily in bacteria and archaea that excises OG paired with cytosine, generating an a apurinic/apyrimidinic (AP) site subsequently processed by BER to restore the G:C base pair [6,7]. If OG escapes MutM-mediated repair, the pro-mutagenic OG:A mispair is generated after replication. MutY removes adenine opposite OG, producing an AP site that is required to regenerate OG:C, thereby allowing MutM another opportunity for lesion removal [8].

MutT, the third component of the GO system, is not a DNA glycosylase. Instead, it is d(OG)TPase that hydrolyzes oxidized d(OG)TPs, preventing their incorporation into DNA by DNA polymerases [9]. Initially, the GO system was described in *Escherichia coli,* but there was substantial evidence of its existence in other prokaryotes and eukaryotes [4]. Today, it is well documented that such a mechanism is present, at least partially, in most genera of each domain of life [10,11]. Particularly, in human cells, the GO system is encoded by the *MUTYH* and *MTH1* genes, whose protein products are MutY and MutT homologs, respectively. Regarding MutM, the human genome does not harbor a homolog which acts directly and efficiently on OG:C lesions. Instead, OGG1 is the human OG DNA glycosylase that belongs to the Helix–Hairpin–Helix (HhH) DG superfamily [11,12]. Figure 1B shows a scheme of the human OGG1- and MUTYH-mediated BER.

Woven within the many years of studying of OGG1 and MUTYH are threads indicating functional roles beyond DNA repair. For example, OGG1 is involved in modulating the expression of several genes involved in immunological response and carcinogenesis [13,14,15]. Telomeres undergoing oxidative stress accumulate OG, ultimately leading to rapid cellular senescence [16], as a consequence of replicative stress mediated by OGG1 and MUTYH [17]. Similarly, MUTYH-initiated BER functions have been shown to act as a molecular switch for tumor suppression, inducing apoptosis in cells burdened with excessive oxidative DNA damage [18,19]. Herein, we aim to summarize what is known about OGG1 and MUTYH transcriptional and posttranslational regulation, as well as what is known about how repair activity is modulated by protein–protein interactions (PPIs). We previously reviewed the structural and biochemical properties of these enzymes as members of the HhH DNA glycosylase superfamily [11].

## 2. OGG1

OGG1 is the main DG in charge of the direct removal of OG opposite C in human cells [11]. OGG1 can also excise the ring-opened lesion 2,6-diamino-4-hydroxy-5-formamidophyrimidine (FapyG) with comparable efficiency [20]. Although both lesions are miscoding, their distinct structures lead to different mutagenic outcomes during DNA replication [21]. OG adopts a rigid planar structure with syn and anti conformers, arising from rotation about the N-glycosidic bond. The *syn* conformer of OG presents its thymine-mimicking Hoogsteen pairing face to the DNA polymerase to mediate A mis-insertion and ultimately GC→TA transversion (Figure 1A). In contrast, the cleavage of the 5-membered ring in FapyG allows for more varied structural conformations rendering different promutagenic outcomes. This includes GC→AT transitions, as well as GC→TA and GC→CA transversions [21,22,23]. The oxidation of G to OG within the genome is estimated to occur with the high frequency of 500 to 1000 lesions cell^−1^ day^−1^ [24] and both OG and FapyG are accumulated at a similar extent when cells are irradiated with UV light [25]. However, FapyG is detected at approximately threefold higher levels than OG in human leukemia cells exposed to ionizing radiation [26,27]. In contrast, hydroxyl radical oxidation under physiological conditions does not produce significant amounts of FapyG [28,29], suggesting that this lesion is unlikely to arise endogenously, but may be a factor to consider in radiation therapy. Several additional DNA glycosylases contribute to the repair of FapyG lesions, including NTHL1, NEIL1, and NEIL3, which process FapyG in distinct DNA contexts [30,31,32,33,34,35]. NEIL1 exhibits negligible activity on OG:C lesions [36,37], underscoring the central role of OGG1 in OG repair.

The importance of OGG1-mediated repair is reflected in its association with multiple pathologies, including metabolic syndromes [38,39], inflammatory diseases [13,40], cancer [41,42], and neurological diseases like Alzheimer’s [43,44] and Parkinson’s [45]. Moreover, OGG1 and MUTYH might play an evolutionary role in maintaining a balanced GC content in eukaryotic genomes [10,46], as defective OG repair imposes strong selective pressure that biases genomes toward increased TA content.

### 2.1. OGG1 as a Transcriptional Modulator

**Figure 2:**
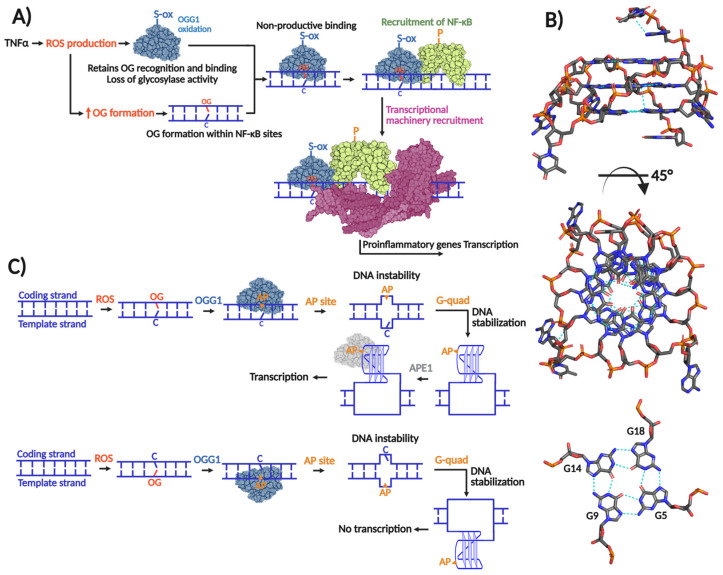
Role of OGG1 in gene transcription. (**A**) Transcriptional induction exerted by OGG1 by non-productive mechanism in which OG recognition is required without glycosylase activity. TNFα-induced ROS production inactivates OGG1’s glycosylase activity probably by means of cysteine oxidation without affecting OG recognition. When OGG1 recognizes and binds OG within proinflammatory gene promoters, it recruits the transcriptional factor NF-κB, which in turn recruits the transcriptional machinery to initiate transcription. (**B**) Transcriptional induction/repression exerted by OGG1 by productive mechanism that implies OG excision and G-quadruplex formation. When OG is formed within the coding strand, its excision by OGG1 forms an AP site causing a concomitant thermodynamically unfavorable form of DNA forcing to adopt a thermodynamically favorable G-quadruplex structure. The AP site in the G-quadruplex is recognized by APE1, which in turn recruits the transcriptional machinery. In the case that the G-quadruplex is formed within the template strand, transcriptional repression results; modified from [60]. (**C**) G-quadruplex structure of human c-MYC promoter solved by NMR (PDB 2LBY; [61]).

OG is unevenly distributed throughout the genome [1,47,48], governed in part by chromatin topology and DNA sequence context. GC-rich sequences, such as those localized in *cis*-regulatory elements of promoters, are the most susceptible to oxidative DNA damage, and therefore, OG accumulation [1,2]. Notably, more than 70% of human promoters are GC-rich regions [49]. Consequently, OG—and by extension OGG1—may exert significant influence over promoter dynamics and gene transcription. Interestingly, OGG1’s influence over promoter activity is mediated by two distinct modus operandi; one relies simply on unproductive OG recognition within promoters, and the other promotes or prevents G-quadruplex formation (Figure 2). Both mechanisms initiate signaling cascades that modulate transcription via transcriptional machinery recruitment.

A series of studies from the Boldogh laboratory established a link between OG formation, OGG1, and inflammatory signaling. Early evidence from the BALB/c mouse models of airway inflammation showed that reduced OGG1 expression attenuates airway hyperresponsiveness, implicating OGG1 in proinflammatory response [50], indicating that OGG1 was participating in proinflammatory responses. OGG1 expression enhances TNFα induced activation of the proinflammatory cytokine CXCL2 [51]. Among the different molecular events triggered by TNFα is an almost immediate (within 30 min) increment of intracellular ROS [52], OG accumulation in GC-rich promoters of proinflammatory cytokine encoding genes [51,53], and release and nuclear translocation of NF-κB, a master transcriptional factor (TF) that regulates inflammation [54]. ChiP analyses revealed enrichment of both OGG1 and NF-κB enrichment at *TNF* responsive promoters; however, OGG1 binding did not coincide with reduced OG levels, indicating that NF-κB recruitment is independent of OG excision [53,55]. Instead, OGG1 enzymatic activity is transiently suppressed following TNFα exposure through reversible cysteine oxidation, as evidenced by recovery with DTT [53]. These findings support a model in which TNFα -induced ROS inactivates OGG1, enabling nonproductive binding at OG-enriched GC-rich promoters. This inactive OGG1 scaffold facilitates recruitment of transcription factors such as NF-κB, thereby promoting proinflammatory gene expression (Figure 2A) [47]. Of note, it was reported that OG is able to modulate gene expression without need of its repair [56]. Moreover, several innate immune cells are hypersensitive to DNA oxidation due to insufficient downstream BER activity, ultimately leading to apoptosis [57,58,59]. These observations highlight critical roles for both OG and OGG1 in shaping inflammatory transcriptional responses and immune cell fate.

The transcriptional activation mechanism that relies on OGG1’s enzymatic activity is associated with G-quadruplex structures. As mentioned, GC-rich promoters are susceptible to formation of OG. Many of these promoters harbor repeating arrays of guanines (G_n≥3_N_1–7_G_n≥3_N_1–7_ G_n≥3_N_1–7_G_n≥3_), which may fold to form three-dimensional DNA structures with stacked quartets of guanines stabilized by Hoogsteen base pairing and potassium ion coordination [62] (Figure 2B). Genome-wide G-quadruplex-seq analyses have identified over 700,000 G-quadruplexes in the human genome [63], of which approximately 10,000 are mapped within promoters and 5′-UTR regions [64]. Moreover, many DNA repair gene promoters are predicted to form G-quadruplex structures, including those from BER genes such as: *NTHL1*, *PCNA*, *FEN1*, *NEIL1*, and *NEIL3* [64,65,66], as well as oncogenes promoters like *KRAS* and *VEGF* [67,68]. Therefore, G-quadruplexes can up- or downregulate repair of oxidative DNA damage, and indirectly, their own formation.

Burrows and co-workers proposed that formation of G-quadruplexes within potential G-quadruplex forming sequences (PQS) requires a series of destabilizing steps [69]. Oxidation of a 5′ G to form OG within the PQS and subsequent OG excision by OGG1 and formation of an AP site combine to destabilize local duplex formation, favoring folding into a G-quadruplex structure. APE1 then recognizes the AP site and recruits transcription factors to initiate transcription (Figure 2C). Notably, APE1’s pro-transcriptional function requires AP-site binding, but not endonuclease activity [70], similar to OGG1 induction of proinflammatory genes. The regulatory outcome of this pathway depends on G-quadruplex positioning; structures formed on the coding strand promote transcription, whereas those on the template strand repress gene expression [60]. Beyond this mechanism, additional roles for OGG1 in transcriptional regulation have been reported [69,71,72].

### 2.2. OGG1 Gene Structure and Regulation

**Figure 3:**
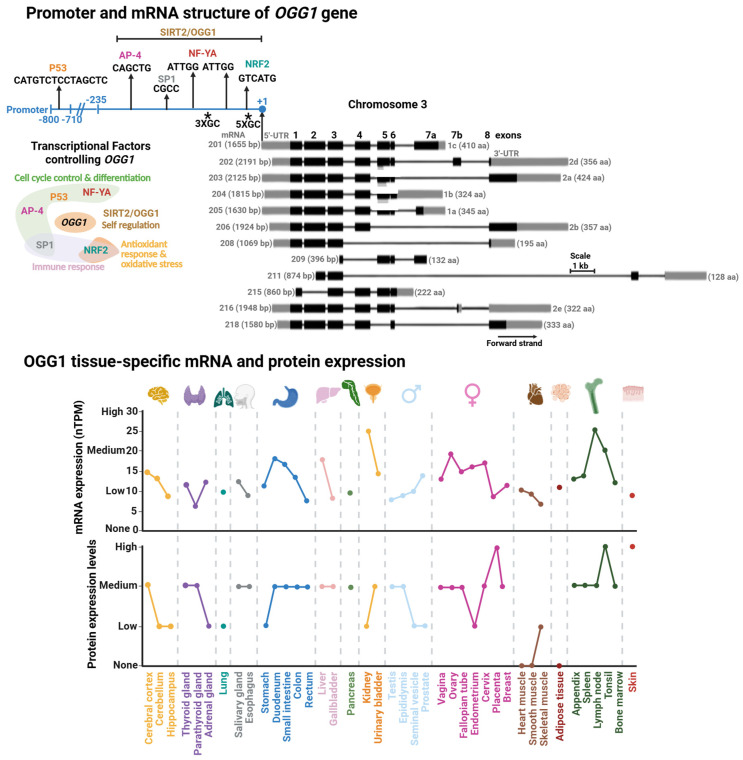
Promoter and mRNA structures of *OGG1* and its tissue-specific mRNA and protein expression. (**Upper Panel**). Transcriptional factor binding sites mapped within *OGG1* promoter. Transcriptional factors (TFs) or proteins controlling *OGG1* expression with their functional annotation are shown. (**Lower Panel**). GC rich regions are indicated with asterisks. Tissue-specific protein and mRNA expression of OGG1. Expression profiles were retrieved from the Human Protein Atlas database [102]. Degree of protein expression is normalized based on None, Low, Medium, and High, as reported in the database. mRNA expression is reported as normalized expression (nTPM) from a consensus dataset combining transcriptomics from the Human Protein Atlas (HPA) and Genotype-Tissue Expression (GTEx) RNA-seq datasets.

The human *OGG1* gene is located in the short arm of chromosome 3 and has a length of 7456 bp. Thus far, 12 distinct functional splice variants have been identified and are produced as a product of the swapping of 8 exons (Figure 3). The most studied isoforms are the α- (1a) and β-isoforms (2a) [73]. Interestingly, all isoforms encode for a mitochondrial localization signal (MLS) at the beginning of the coding region of the mRNA. The α-variants also contain a nuclear localization signal (NLS) at the 3′-end [74]. This means that the NLS is the dominant localization signal for OGG1 intracellular localization.

#### 2.2.1. OGG1 Promoter and Transcriptional Regulation

Initially OGG1 was thought to be a housekeeping gene with relatively consistent expression levels maintained throughout the cell cycle [75]. However, tissue-specific expression data from the Human Protein Atlas reveal substantial variability in OGG1 levels, with particularly high expression in the kidney and lymph nodes (Figure 3, [76]). For instance, in kidney cells, the cell cycle control and cell proliferation processes are under tight regulation, with growth rates less than 1% [77] and a particular arrest in G1/0 of the cell cycle [78]. The organ’s high energetic demand leads to persistent oxidative stress [79] resulting in progressive OG accumulation over the life-span [80]. These observations suggest that OGG1 plays a critical role in removing OG during periods of cell cycle arrest, thereby preventing the accumulation of G:C→T:A transversion mutations prior to DNA replication.

The *OGG1* promoter region was initially studied by Dhénaut and colleagues (2000) [75]. They identified that the promoter region up to position −135 is the minimum active component required to maintain transcription. Moreover, they identified several putative transcriptional factor (TF) binding sites, including sites for SP1 and the nuclear respiratory factor (NRF1; Figure 3). The latter is a specialized TF which regulates antioxidant genes and response to oxidative stress [81]. Further studies demonstrated that downregulation of NRF1 reduces OGG1 expression, establishing NRF1 as a key transcriptional regulator of OGG1 [82]. Disruption of this regulation has been linked to impaired handling of oxidative DNA damage in diabetes [82] and estrogen-induced breast cancer [83]. The SP1 binding site was mapped in a segment containing positions −474 to −431 within the *OGG1* promoter [84]. SP1 regulates genes that control cell cycle control, apoptosis, hormonal activation, and other processes [85]. Notably, cadmium exposure suppresses SP1 binding at the OGG1 promoter, leading to reduced OGG1 expression [84]. Cadmium is a widespread environmental carcinogen that induces chromosomal aberrations, DNA stand breaks, and OG accumulation [86,87]. Thus, the inhibition of SP1 binding to the *OGG1* promoter might play an important role in Cadmium-induced DNA damage and carcinogenesis.

Another set of TFs identified as regulators of *OGG1* are the Nuclear Transcription Factor Y-A (NF-YA) [88,89], AP-4 [90], and p53 [91]. Of note, the tumor suppressor p53 regulates different DNA repair pathways, either indirectly or directly [92,93] including several BER genes, such as *MUTYH* [18], *APE1* [94], and Polβ [95]. These transcription factors regulate cell-cycle progression and their dysfunction is linked to colorectal, gastric, and hepatic cancers [96,97,98]. Recently, it has been reported that OGG1 regulates its own expression [99]. Geng et al. demonstrated that OGG1 is recruited to and physically binds its own promoter, a region which spans from position −297 to +3, to increase *OGG1* expression and OG repair. This recruitment is enhanced by OGG1’s interaction with SIRT2, an NAD^+^-dependent sirtuin deacetylase that targets histones H3 and H4 [100,101]. However, the OGG1-SIRT2 interaction and induction of *OGG1* transcription are independent of acetylation/deacetylation. Instead, it relies on upstream events such as SIRT2 phosphorylation in an ATM/ATR-dependent manner. Corroborating this finding, the OGG1-SIRT2 interaction is enhanced in cells subjected to oxidative stress [99], suggesting that oxidative DNA damage serves as the initiating signal for OGG1 transcriptional activation.

#### 2.2.2. Epigenetic Control of OGG1

As a GC rich promoter including several SP1 binding sites [75,84], the *OGG1* promoter is epigenetically active and linked to cellular responses to pesticide exposure, arsenic genotoxicity, and several diseases. For instance, methyl parathion, an oxidizing organophosphate pesticide that induces oxidative stress in germ cells [103] causes global hypomethylation and an increase in promoter-specific methylation of *OGG1* at two CpG sites [104]. Likewise, Wang et al. (2021) showed that arsenic exposure in human bronchial epithelial (HBE) cells induces oxidative stress with concomitant inhibition of TET-mediated DNA demethylation, resulting in OGG1 hypermethylation and reduced protein expression [105]. Another physiological challenge that affects *OGG1* methylation is the prolonged exposure to estrogen 17 beta-estradiol, which is associated with breast cancer [106]. In a prospective longitudinal cohort study including 582 male participants from the Boston, USA, the DNA methylation levels of different genes in blood leukocytes were sampled over the course of nine years. The results indicated that the *OGG1* promoter region was methylated, which is associated with certain types of cancer, including prostate [107].

Oxidative DNA damage and OG accumulation are biomarkers for neurodegeneration and aging [80,108,109]. In fact, the low expression of OGG1 and MTH1 is a hallmark of Alzheimer’s disease (AD) [110,111]. These data suggest that the partial dysfunction of the GO system exacerbates the progression of AD. Interestingly, there is evidence that this correlation might have an epigenetic origin. Italian and Chinese late-onset AD cohorts reported modest but detectable changes in OGG1 promoter methylation in peripheral blood cells, including *APOE ε4* carriers [112,113]. However, in a Polish population, OGG1 promoter methylation accompanied by reduced mRNA expression in AD patients [114]. Although these differences may reflect ethnic or genetic variability [115], altered methylation of the OGG1 promoter emerges as a recurring feature associated with AD and other diseases.

### 2.3. OGG1 Protein Structure and Regulation

**Figure 4:**
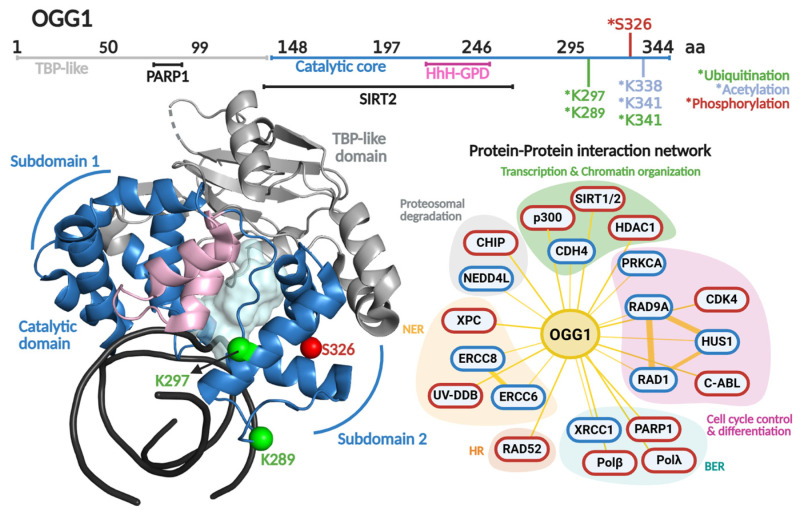
OGG1 primary and quaternary structures in complex with DNA and their protein–protein interaction network. On top, a scheme of the amino acid sequence is shown highlighting (in different colors) each functional domain. Residues subjected to posttranslational modifications and regions involved in protein–protein interactions are indicated. Ubiquitination, acetylation and phosphorylation are indicated with asterisk in green, clear blue and red colors, respectively. The crystallographic structure of OGG1 (PDB 1FN7 [116]) is shown in different colors for each functional domain. The catalytic cavity is shown in cyan and the DNA in black. The protein–protein interaction network shows protein partners for OGG1 based on the BioGRIG database (Blue, [122]) and based on literature (Red). The protein partners found in BioGRID were filtered based just on physical interactions. The figure of OGG1 structure was generated in PyMOL version 3.0.4.

In 2001, the Verdine laboratory reported the first crystal structure of OGG1 [116]. Distinct from other HhH enzymes, OGG1 is a modular DG composed of two domains; the HhH-GDP superfamily domain and the TATA-binding protein (TBP)-like domain. The latter domain does not make contact with the DNA and has been proposed to serve as a scaffold for PPIs, based on being the locus for binding of the DNA break sensor Poly (ADP-ribose) polymerase 1 (PARP-1) [117]. The HhH-GDP domain carries out OG recognition, DNA engagement, and catalysis. It comprises two α-helical subdomains linked by the featured HhH motif (Figure 4). These subdomains flank the catalytic pocket where catalytic residues Asp268 and Lys249 participate in the excision of OG opposite C, and strand scission at the 3′ end of the produced AP site via β-elimination [116].

Regulation of OGG1 at the protein level occurs via posttranslational modifications (PTM) and PPIs. Particularly, PTMs (phosphorylation, acetylation, ubiquitination) are able to control enzymatic activity, protein stability, intracellular localization, and even PPIs [118] (Table 1). This type of protein regulation has been described as pivotal in maintaining genomic integrity [119].

#### 2.3.1. Ubiquitination

Several studies have indicated that OGG1 may be ubiquitinated. An initial report showed that OGG1 is ubiquitinated in response to hyperthermia, namely in Hela cells at 42 °C [120]. In this study a marked decrease of OGG1 activity was found after 2 h of thermic treatment. The decrease in OGG1 DG activity was explained by two means; (1) heat-induced misfolding, and (2) a diminishment in OGG1 protein levels mediated by proteasomal degradation. The authors identified the C-terminus of HSC70-interacting protein (CHIP) as the E3 ligase involved in the ubiquitination of OGG1 in hyperthermic conditions. However, the residues of OGG1 subjected to ubiquitination were not identified. A study by Parson and co-workers identified NEDD4L as an E3 ubiquitin ligase targeting OGG1 specifically at residue Lys341 [121]. Interestingly, in cellulo studies where NEDDL4 downregulation is carried out in U2O2 cell lines demonstrated that OGG1 protein stability is enhanced under ionizing radiation-induced oxidative stress. Moreover, OGG1 ubiquitination inhibits its glycosylase/lyase activity in vitro. The authors correlated the prolonged OGG1 stability and the enhanced DG activity with poor cell survival post irradiation.

Ubiquitination has also been suggested to mediate the repair of OGG1-DNA crosslinks implicated to form in cells [123]. DNA–protein crosslinks are potent contributors to genome instability, aging, and cancer [124]. The OGG1-DNA crosslinks form by Schiff base formation between Lys residues of OGG1 and 3′-phospho-α,β-unsaturated aldehyde (3′UA) intermediates arising from OGG1-mediated β-elimination at the AP site product of OGG1 glycosylase activity (Figure 1) [11,125]. Lys249 can form a Schiff base with the 3′-UA, and in vitro, the Schiff base can be detected via reduction with NaBH_4_ treatment to form a stable DNA–protein crosslink [12,125]. OGG1-DNA crosslinks can be repaired by two different mechanisms, both mediated by ubiquitination: NER or homologous recombination [126]. Specifically, OGG1 polyubiquitination of Lys341 via Lys48 ubiquitination leads to proteasomal degradation and resolution of the lesion via NER. However, when OGG1 is polyubiquitinated by Lys63 ubiquitination, the lesion is resolved via homologous recombination in a proteasome-independent manner.

**Table 1:** Posttranslational modifications (PTM) and protein–protein interaction (PPI) controlling OGG1.

PTM	Site	Effect [Reference]
Ubiquitination	K341	↓ glycosylase/lyase activity [121], ↑ proteasomal degradation [126]
	K289	Unknown [127]
	K298	Unknown [128]
Phosphorylation	S326	↑ glycosylase activity [129]
Acetylation	K338/K341	↑ turnover [130,131]
**PPI**	**Effect [Reference]**	
Polβ	↑ turnover [132]	
Polλ	Unknown [132]	
XPC	↑ turnover [133]	
UV-DDB	↑ turnover [134]	
RAD52	↑ turnover [135]	
PARP1	↓ glycosylase activity [117]	
XRCC1	↑ glycosylase and lyase activity [136]	
RAD9-RAD1-HUS1	↑ glycosylase and lyase activity [137]	

Up-arrow (↑) and down-arrow (↓) symbols indicate an enhancement or diminishment of the referred biochemical parameter.

OGG1’s Lys289 [127] and Lys297 [128] have also been identified as targets for ubiquitination through high-accuracy mass spectrometry approaches. However, there is no characterization of the biochemical and cellular effects of ubiquitination at these residues. Nonetheless, ubiquitination clearly serves as a regulatory mechanism for OGG1 activity, facilitating the removal of potentially lethal intermediates generated during BER. This underscores the importance of these PTMs in fine-tuning repair and preventing futile DNA processing.

#### 2.3.2. Phosphorylation

The first PTM reported for OGG1 was serine phosphorylation, described in 2002 [138]. Although the modified residue was not identified, chromatin-associated OGG1 was shown to co-precipitate with protein kinase C (PKC), and in vitro phosphorylation experiments demonstrated that this PTM was carried out by the α, β, and γ isoforms of PKC. This modification does not impact OGG1 glycosylase activity, but instead regulates its nuclear localization [138]. Phosphorylated OGG1 associates with chromatin, while unphosphorylated protein localizes to the nuclear matrix. OGG1 is found in both compartments during interphase, but association to condensed chromatin is specific to mitosis. These findings suggest that PKC-mediated phosphorylation spatially regulates OGG1, potentially facilitating OG detection and repair across distinct chromatin states and phases of the cell cycle.

Additional kinases have been reported to phosphorylate OGG1, including the serine kinase CDK4 and threonine kinase c-Abl [129]. Although the OGG1 residues these kinases target remain unknown, the in vitro phosphorylation carried out by Cdk4 enhanced OGG1 glycosylase activity 2.5-fold. However, phosphorylation by c-Abl did not cause any changes in biochemical behavior. In silico analysis has identified Ser326 as a potential phosphorylation site. Notably, this residue corresponds with a cancer associated polymorphism (S326C) [139,140,141], whose functional relevance is underscored by defects in nuclear localization [142] and increased genomic instability in homozygous cells [143].

#### 2.3.3. Acetylation

OGG1 acetylation was first reported by the Mitra laboratory in 2006, showing that it was acetylated in HeLa cells [130] with an 2.5-fold increase in response to oxidative stress. The acetyltransferases p300 was identified as being responsible for OGG1 acetylation, mainly at Lys338 and Lys341. In vitro studies demonstrated that this PTM significantly increases OGG1 turnover mediated by APE1. Likewise, an interaction of the deacetylase HDAC1 interaction with OGG1 was detected in immunoprecipitation experiments [130]. Lys338 and Lys341 acetylation have been observed in age-related cataracts (ARCs), a common eye disease in the elderly [144]. Immunoprecipitation and siRNA assays confirmed that p300 was the major acetyltransferase in lens epithelium cells and demonstrated that the deacetylase SIRT1 actively removes acetyl groups from OGG1. OG accumulation in an ARC could result from an imbalance of OGG1 interactions with p300 and SIRT1, favoring deacetylation and reduced OG removal. Similar regulatory patterns have been observed in human skeletal muscle following exercise [130] and in rat hippocampal cells [145].

In contrast, SIRT3 is an NAD^+^-dependent deacetylase with mitochondrial activity. Indeed, mitochondrial OGG1 physically interacts with and is deacetylated by SIRT3, a modification which stabilizes OGG1, enhances OG excision, and protects genomic integrity by limiting apoptosis during oxidative stress [146]. These findings collectively underscore the importance of acetylation in regulating OGG1 activity.

### 2.4. OGG1 Regulation by Protein–Protein Interactions

OGG1’s PPIs have been a topic of interest since early 2000 [147,148,149]. In Figure 4, an updated PPI network of OGG1 is displayed, highlighting protein partners involved in different cellular processes including transcriptional regulation, chromatin remodeling, proteasomal degradation, cell cycle control, and cellular differentiation. As expected, OGG1 interacts with AP-site signaling proteins involved in BER (PARP1 and XRCC1), as well as with NER through its interaction with ERCC8 and ERCC6. This complex and broad PPI network strongly suggests that expression and activity of OGG1 is fine-tuned by different cellular processes and vice versa. In this section, we will delineate an updated version of OGG1’s PPI network, focusing on recent reports and newer interpretations.

#### 2.4.1. Regulation of OGG1 Turnover

Most DGs bind their product (AP-containing DNA) more tightly than their substrate in vitro [148]. Particularly, OGG1 forms a stable complex with DNA with a half-life of 18 min [150]. This stable complex is due to OGG1’s 400-fold tighter affinity for AP: C-containing DNA than its substrate, OG:C (*K_d_* < 0.005 vs. 2 nM, respectively) [151]. Such differences in binding renders a markedly slow turnover (*k*_3_ = 0.04–0.08 min^−1^) [34,152]. In stark contrast is APE1’s turnover, with a rate of at least 2.7 min^−1^ [153]. This suggests that OGG1’s turnover is the rate limiting step in OG processing, and potentially a regulatory point for BER. OGG1’s turnover is stimulated by other DNA repair enzymes like APE1 [154,155,156] and NEIL [34]. Interestingly, despite OGG1 turnover stimulation, there is no physical interaction reported with APE1 nor NEIL1. The mechanism proposed implies a transient release of the AP site by OGG1 that allows its rapid and opportunistic binding and processing by APE1, preventing retrograde OGG1 binding [157]. The Zharkov Lab demonstrated that Polβ and OGG1 interact with an affinity of 580 nM (*K_d_*) and such interaction favors OGG1’s turnover by means of disrupting OGG1-DNA complexes, potentially by displacement of OGG1 from its product by passive diffusion of Polβ [158].

Other PPI reported to promote OGG1 turnover are Xeroderma pigmentosum C (XPC) and the UV-damaged DNA binding (UV-DDB) NER proteins. Both participate in recognizing UV-induced lesions like cyclobutene pyrimidine dimers (CPD) and (6-4) photoproducts, and recruitment of downstream NER proteins to the repair site [133,134]. Far western analysis demonstrated that XPC and OGG1 physically interact, resulting in enhanced OGG1 turnover [133]. More recently, the Van Houten Lab demonstrated that UV-DDB has 2-fold higher affinity for AP sites than OGG1 [134]. Consequently, UV-DDB promotes OGG1’s turnover by competing for the AP site [157]. Similar results were reported for MUTYH (see Section 3.4.3). Another partner for OGG1 is the homologous recombination protein RAD52. This interaction displays contrasting biochemical effects with inhibition of RAD52, while OGG1 turnover is stimulated [135]. The interaction of OGG1 with RAD52 suggests a regulatory mechanism of some sort but the details and implicated remain to be clarified

#### 2.4.2. OGG1 Interactions with DNA Strand Break Signaling Proteins

As stated, OGG1 interacts through its TATA binding-like domain with protein the C-terminal domain of PARP-1 in vitro and in cellulo [117]. PARP-1 plays a role in repairing ssDNA breaks through PARylation of itself and surrounding proteins when bound to ssDNA breaks derived from AP sites processed by APE1. Enhanced PARP-1 PARylation activity leads to OGG1 inhibition. This inhibition only occurs upon incubation with active PARP-1 in the presence of its cofactor NAD^+^. Although the mechanisms of OGG1 PARylation are not completely understood, its role in modulating OG repair may help explain the etiology of several OGG1-associated pathologies.

The X-ray repair cross-complementing protein 1 (XRCC1), functions as a molecular scaffold that interacts with a variety of DNA repair proteins to signal and accelerate the repair of ssDNA breaks [159]. Marsin et al. (2003) reported a physical and functional interaction between OGG1 and XRCC1 which enhances up to 3-fold and 2-fold the OGG1 glycosylase activity and Schiff base intermediate formation associated to lyase activity, respectively [136]. Interestingly, XRCC1 and APE1 have a synergistic effect on OGG1 glycosylase activity. A structural mapping of the interaction between XRCC1 and OGG1 showed that two regions participate in the interaction, the BRCT1 and a hinge region between the N-terminal domain and BRCT1 of XRCC1. Surprisingly, APE1 binds to both sites, as well. These results suggest that XRCC1 functions as a facilitator of the AP-site hand off between OGG1 and APE1, enhancing the processivity of at least the first two reactions in the coordinated repair of OG.

#### 2.4.3. OGG1 Interaction with Cell-Cycle Control Proteins

Both OGG1 and MUTYH have been reported to interact with the 9-1-1 checkpoint complex [137,160], a heterotrimer comprising Rad9, Rad1, and Hus1 subunits, adopting a donut-like structure similar to PCNA [161]. The 9-1-1 complex senses stalled replications forks produced by ssDNA breaks formed directly by DNA damage or as intermediates of DNA repair events. Once the complex recognizes these abortive replication sites, the DNA damage checkpoint response is to arrest the cell cycle [162].

Park et al. (2009) characterized the in cellulo and in vitro interaction between OGG1 and the 9-1-1 complex [137]. In HEK293 cells treated with H_2_O_2_, OGG1 and RAD9 co-localized in the nucleus, suggesting that the OGG1-9-1-1 complex is formed following OG formation. Enzymatic studies demonstrated that OGG1 does not bind a specific subunit of the 9-1-1 complex, but instead, each monomer stimulates OG excision and Schiff base formation to a similar extent. However, the full 9-1-1 complex rendered major OG repair in vitro and in vivo. OGG1, as a bifunctional DG, is able to generate ssDNA breaks, so the non-specific binding of OGG1 to the 9-1-1 complex might be triggered as a product of ssDNA break formation.

## 3. MUTYH

MUTYH is a peculiar DG, as it excises an undamaged adenine erroneously base pairing with the OG lesion, thereby preventing the formation of stable T:A base pairs. The recombinant MUTYH enzyme also excises A opposite G, although with reduced efficiency compared to the OG:A substrate [163]. However, experiments in *Escherichia coli* and HEK293FT cells showed that G:A is not efficiently processed by MUTYH in a cellular context [164,165], suggesting that OG:A is the preferable substrate. Human MUTYH displays base excision activity for 2-hydroxyadenine opposite each canonical base and OG [166], as well, but the biological significance of 2-hydroxyad enine repair remains unclear.

Another peculiarity of MUTYH is its narrow substrate specificity. Whereas most DNA glycosylases are promiscuous, MUTYH displays a strict preference for OG:A. For example, NTHL1 and AlkA are each capable of repairing more than nine DNA lesions [11]. Structural analyses of MutY/MUTYH and other HhH-DGs have shown that the strict preference of MUTYH for OG:A is due to the tight fit of adenine inside the active site, as well as a broad H-bond network between residues involved in A and OG recognition [11]. Moreover, most DGs interact primarily with the lesion-containing strand, but MUTYH makes extensive contacts with both the A- and OG-containing DNA strands [11,167].

### 3.1. MUTYH as Mutagenesis and Tumor Suppressor During Oxidative Stress

The importance of MUTYH in DNA repair is accentuated by an increased risk of polyposis and colorectal cancer (CRC) predisposition in individuals with inherited *MUTYH* variants, in an autosomal recessive condition known as MUTYH-associated polyposis (MAP) [168,169,170]. Individuals harboring biallelic germline mutations display an increased risk of developing colorectal cancer. Moreover, MAP patients have an increased risk of developing extracolonic cancers such as ovarian, bladder, breast, and others [169]. Although <1% of colorectal cancers are related to biallelic *MUTYH* mutations, about 1 to 2% of the worldwide population are carriers of monoallelic *MUTYH* mutations [171]. Of note, reports have been made proposing that monoallelic mutations in *MUTYH* itself or in combination with other pathologies might be a driver of the carcinogenic process [172,173,174,175,176]. Although the mechanism by which monoallelic mutations in *MUTYH* become pathologic remains unknown, these studies strongly suggest that the spectrum of diseases and underlying molecular mechanism in which mutated *MUTYH* participates is beyond MAP.

MAP arises from inherited loss-of-function mutations leading to increased G:C→T:A transversions. The mutational burden elevates the likelihood of oncogenic alterations in genes like *APC* and *KRAS* [177]. A role of MUTYH in tumor-suppressive signaling during acute oxidative stress has also been suggested [18,178]. For instance, *MUYTH*-Knockout mice showed the highest increase (5.3-fold) in tumorigenesis events (particularly in the colon) and up to a 70-fold increase under KBrO3-induced oxidative stress [41,179,180]. This suggests that MUTYH is required to attenuate tumorigenesis.

**Figure 5:**
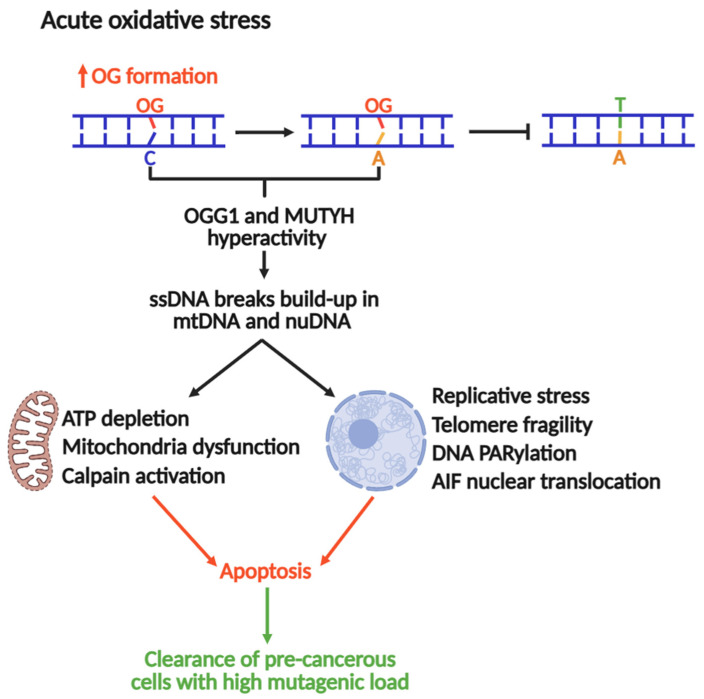
MUTYH and OGG1 as drivers of genomic instability and apoptosis to suppress tumorigenesis under acute oxidative stress.

The Nakabeppu laboratory demonstrated that selective accumulation of nuclear or mitochondrial OG reduced cell viability in response to menadione-induced oxidative stress, indicating that OG accumulation in each organelle triggers cell death distinctly [19]. Nuclear OG-induced cell death requires PARP activity and concomitant nuclear translocation of Apoptosis-Inducing Factor (AIF), which promotes chromatin condensation, DNA fragmentation, and cell death [19,181]. In contrast, mitochondrial OG accumulation leads to functional and morphological degeneration [19]. Consistent with these outcomes, oxidative stress is accompanied by marked activation of calpain, a calcium-dependent protease that contributes to apoptotic cell death [182]. *MUTYH* downregulation significantly diminishes ssDNA breaks and cell death during oxidative stress, concomitant with abrogation of AIF and calpain activation. These findings suggest that excessive build-up of OG:A in cells might lead to futile MUTYH-mediated repair, which can be cytotoxic. In this context, MUTYH shifts from maintaining the genome to a pro-apoptotic trigger, eliminating cells at high risk of acquiring OG-induced transversions (Figure 5).

Recent work published by the Opresko lab suggests that OG in telomeres causes replicative stress and an excess of ssDNA breaks, inducing telomeric fragility [16]. A follow-up study associated this replicative stress to OGG1 and MUTYH repair activities, where *MUTYH* and *OGG1* knockouts in fibroblasts rescued OG-induced telomere fragility [17]. Therefore, under conditions of acute oxidative stress, the repair of OG produces intermediates such as ssDNA breaks or DG-crosslinks (as reported for OGG1) [126], which stall DNA replication, a major source of genomic instability and cytotoxicity. Consequently, MUTYH-mediated DNA repair and pro-apoptotic activities should be strictly regulated to control the mutagenic load and cellular fate. Moreover, the full understanding of this mechanism might be a potential avenue for the development of therapies against MAP and other types of cancers.

### 3.2. MUTYH Gene Structure and Regulation

**Figure 6:**
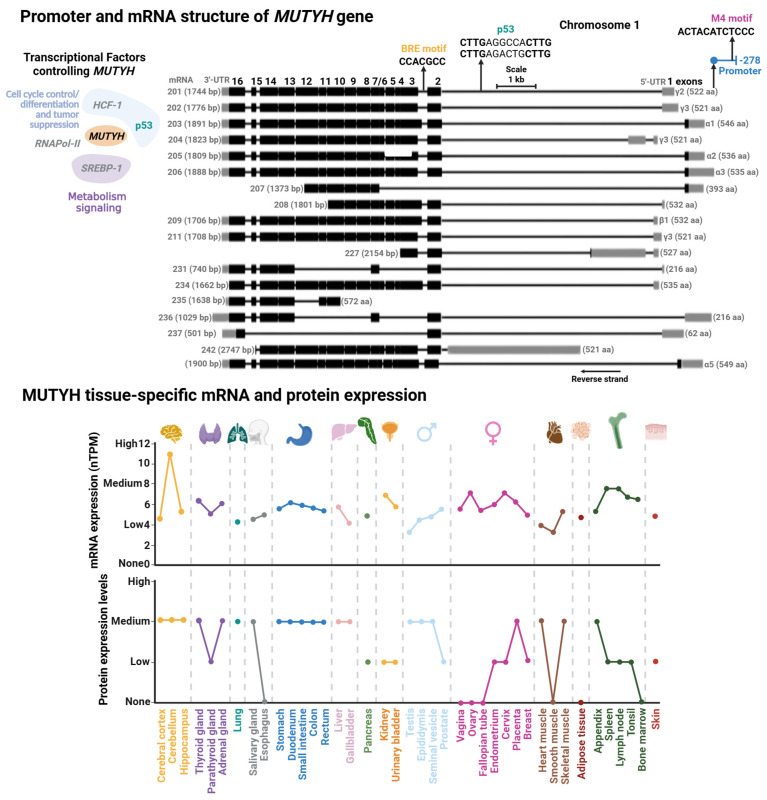
Promoter and mRNA structures of *MUTYH* and its tissue-specific mRNA and protein expression. (**Upper Panel**) Transcriptional factor binding sites mapped within *MUTYH* promoter. Transcriptional factors (TFs) controlling *MUTYH* expression with their functional annotation are shown. TFs shown in gray are suggested according to TFs-binding M4 motif [183]; however, there is no direct evidence for their regulation of *MUTYH.* (**Lower Panel**) Tissue-specific protein and mRNA expression of MUTYH. Expression profiles were retrieved from the Human Protein Atlas database [102]. Full description of this analysis is shown in Figure 3.

The human *MUTYH* gene is mapped on the short arm of chromosome 1 and has a length of 11.2 kb with 16 exons (Figure 6). Currently, 18 transcripts have been identified, clustered into three main types: α, β, and γ. The main difference among these transcript variations are the first exons where the 5′ Untranslated Region (UTR) length differs. Namely, in the case of the α transcripts, the first exon also harbors a mitochondrial localization peptide. These transcripts derive from alternative splicing and different transcription start sites modulated by transcription factors [183] (discussed below). A shared feature in most *MUTYH* mRNA is the presence of exons 4 through 15 and a Nuclear Localization Peptide at the beginning and end of the mRNA. Of note, although mitochondrial isoform α have three protein localization signals (two NLS and an MTL), they are mainly mitochondrial MUTYH isoforms. In contrast with OGG1, MLS is the dominant localization signal in MUTYH [178]. The isoforms α3, β3, β5, and γ3 are the most abundant transcripts of MUTYH [18].

#### 3.2.1. MUTYH Promoter and Transcriptional Regulation

Regulatory elements controlling MUTYH expression were first characterized in 2014. Oka et al. showed that nuclear MUTYH-β transcripts are regulated by the tumor suppressor p53 by two p53 response elements located within the first intron of the *MUTYH* gene [18] (Figure 6). More recently, the Plotz laboratory identified two regulatory elements within the *MUTYH* promoter: an M4 and a B-responsive element (BRE) [183]. M4 is the cognate site of SREBP-1 and HCF-1 TFs, which participates in genetic regulation on sterol metabolism [184], and hematopoietic and embryonic stem cells [185]. Mutational studies demonstrated that the M4 motif functions as a global regulator of most *MUTYH* mRNAs [183]. The BRE motif is a core promoter element associated to RNA polymerase II-dependent transcription. Mutational analysis and location of the transcriptional start site of the BRE motif and GC-box suggest that these elements regulate nuclear MUTYH-β and mitochondrial MUTYH-α mRNAs, respectively.

#### 3.2.2. MUTYH Epigenetic Control

The genetic expression of *MUTYH* has been explored in different cell lines, as well as in vivo and ex vivo with different genotoxic stressors and pathological conditions [114,186,187,188,189]. A common feature in most of these studies is that *MUTYH* mRNA expression is tissue- and cell cycle-dependent. *MUTYH* is highly expressed in the brain, mildly expressed in the respiratory system, skin, and reproductive tissue, and low expression in the gastrointestinal system (Figure 6). It has been reported that organellar *MUTYH* transcripts are also differentially expressed in different tissues and different stages of the cell cycle [190,191,192]. For instance, the nuclear MUTYH mRNA-β is upregulated in proliferating cells, while variant α is mainly expressed in post mitotic human cells [191]. The association of *MUTYH* expression and cellular proliferation is consistent with OG:A mismatches as replication-associated DNA lesions. Highly proliferative cells, such as those derived from the reproductive system, are exposed to more replicative stress than low proliferative cells, such as muscle cells, correlating with high metabolic demand and ROS production in highly proliferative cells [193,194].

The variable expression profile of *MUTYH* suggests tight tissue-specific regulation to ensure appropriate OG:A repair. Any perturbation that leads to expression changes might trigger adverse consequences for cellular function, as discussed above. This suggests that epigenetics might be a strong regulatory element of *MUTYH* expression. However, contrary to *OGG1,* the regulatory regions of *MUTYH* characterized so far are not particularly GC-rich, and the few sites identified are not regulatorily active [183]. Additionally, to our knowledge, epigenetic control of *MUTYH* has not been explored. Nonetheless, several studies have implicated dysregulation of *MUTYH* epigenetic control in pathologies like sporadic colorectal cancer [195,196,197].

### 3.3. MUTYH Protein Structure and Regulation

**Figure 7:**
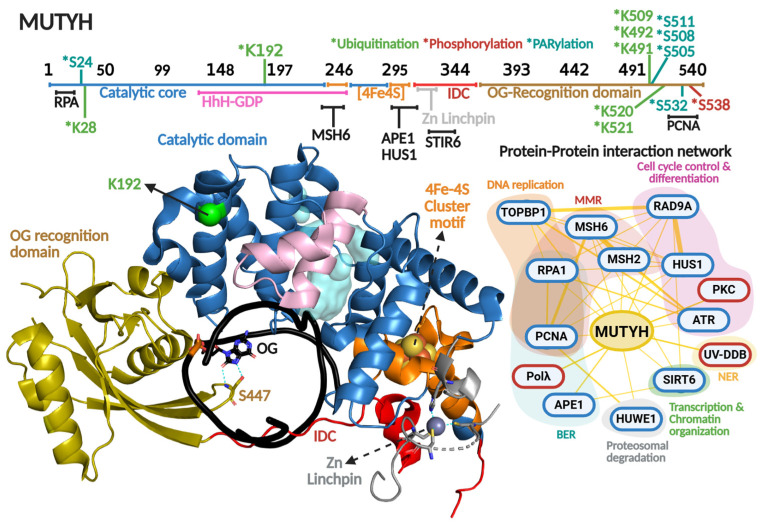
MUTYH primary and quaternary structures in complex with DNA and their protein–protein interaction network. On top, a scheme of the amino acid sequence is shown highlighting (in different colors) each functional domain. Residues subjected to posttranslational modifications and regions involved in protein–protein interactions are indicated. Ubiquitination, PARylation and phosphorylation are indicated with asterisk in green, clear blue and red colors, respectively Crystallographic structure of MUTYH (8FAY [167]) is shown in different colors for each functional domain. Catalytic cavity is shown in cyan and the DNA in black. Zn linchpin motif of MUTYH is superimposed from mouse MUTYH structure (PDB 7EF8 [166]). Protein–protein interactions show protein partners for MUTYH based on the BioGRIG database [122] (Blue edge) and based on the literature (Red edge). Protein partners found in BioGRID were filtered based just on physical interactions. Figure of MUTYH structure was generated in PyMOL version 3.0.4.

OG recognition and adenine excision by MUTYH are highly coordinated [198]. The enzyme possesses two distinct domains: the catalytic domain and the OG recognition domain (Figure 7). The OG recognition domain finds the OG moiety using a region known as the FSH loop, which contains highly conserved Phe, Ser, and His residues (F446, S447, and H448 in relation to MUTYH human isoform α5). The OG within the OG:A mismatch adopts the *syn*-conformation, in contrast to OG or G in OG:C/G:C base pairs that adopt canonical *anti*-conformation of Watson–Crick base pairs [199,200,201,202]. Several studies have shown that bacterial and human MUTYH detect 2-amino group of OG that is exposed in the major groove only in OG-*syn*:A-*anti* base pairs [164,203]. Upon OG:A detection, MutY rotates the OG to the *anti*-conformation, where the 8-oxo and 7-amino moieties are located in the major groove, stabilized with H-bond contacts with the S447 within the FSH loop [198].

Other important structural and functional components of MutY/MUTYH homologs are the coordination of a [4Fe-4S] cluster and zinc ion. The [4Fe-4S] cluster is a cofactor present in bacterial, archaeal, and eukaryotic MutY homologs, while the Zn coordination is exclusively conserved amongst mammalian MUTYH homologs [11]. Both cofactors have been described as important elements in DNA repair, despite not being involved directly in catalysis [167,204,205,206,207]. The [4Fe-4S] is coordinated by four cysteines within the [4Fe-4S] cluster motif, which is part of the catalytic domain, and is involved in DNA lesion localization/recognition and protein stability. One of the most outstanding functional roles of this cofactor is its DNA-mediated redox activity, which modulates MUTYH affinity for DNA and has been proposed to direct the quest for DNA lesions within genomes of MUTYH and other [4Fe-4S] cluster-containing DGs such as NTHL1 [208,209,210]. More recently, we solved the X-ray crystallographic structure of the human MUTYH-DNA complex, and along with biochemical profiling of cancer associated variants, coevolutionary and molecular dynamic analyses, we discovered that the [4Fe-4S] cluster allosterically regulates the positioning and protonation of the catalytic Asp236 residue required for adenine excision [167,211]. Therefore, the [4Fe-4S] cluster is a multifaceted cofactor in MUTYH. The Zn is coordinated by three cysteines and one histidine to form the zinc linchpin motif within the interdomain connector (IDC) that structurally connects the catalytic domain and OG recognition domain of MUTYH [166]. The presence of the Zn linchpin, along with a longer IDC region (up to 6-fold longer than bacterial MutY homologs) has been described as a scaffold for PPIs, as detailed below (Figure 7 and Table 2).

#### 3.3.1. Phosphorylation

The first reports of MUTYH phosphorylation were by Gu and Lu in 2001. They reported that cell extracts from H2009 cell lines showed a marked reduction in MUTYH activity after dephosphorylation treatment [212]. Interestingly, the dephosphorylation effect on MUTYH activity was greater with G:A vs. OG:A-containing DNA substrates (50- vs. 4-fold reduction), suggesting that MUTYH activity and specificity is regulated by this type of PTM. In 2003, Parker et al. showed that defects in MUTYH phosphorylation are responsible, at least partially, for diminished OG:A repair in cell extracts of colorectal cancer cell lines [213]. Through the use of co-immunoprecipitation and in vitro phosphorylation assays, the protein kinase PKC was identified as the kinase responsible for MUTYH phosphorylation (Figure 7). In 2010, the David Lab identified that residue Ser538 is phosphorylated within the PCNA binding region [214]. Mutation to Alanine at this position renders lower protein stability than the WT and S538D phosphomimic mutation. The mutants exhibited a 10-fold reduction in binding affinity for an uncleavable adenine analog opposite OG. Because the phosphorylation site maps to the PCNA-binding region, these biochemical defects suggest that Ser538 phosphorylation is critical for OG:A recognition and coupling MUTYH to DNA replication.

**Table 2:** Posttranslational modifications (PTM) and protein–protein interaction (PPI) controlling MUTYH.

PTM	Site	Effect [Reference]
Phosphorylation	S538	↑ protein stability [214]
Ubiquitination	K28/K192	Unknown [215,216]
	K491/K492/K509/K520/ K521	↑ proteasomal degradation [217] ↓ nuclear translocation [217]
PARylation	S24/S511/S505/S508/S532	Unknown [218]
**PPI**	**Effect [Reference]**	
MutSα	↑ glycosylase activity (at low [MutSα]) [219] ↓ glycosylase activity (at high [MutSα]) [219]	
PCNA	Couples MUTYH repair to DNA replication [220]	
RAD9-RAD1-HUS1	↑ glycosylase activity [160,221]	
APE1	↑ protein:DNA complex formation [222]	
SIRT6	↑ glycosylase activity [223]	
UV-DDB	↑ turnover [224]	

Up-arrow (↑) and down-arrow (↓) symbols indicate an enhancement or diminishment of the referred biochemical parameter.

#### 3.3.2. Ubiquitination

MUTYH harbors seven Lys residues mapped as ubiquitination sites: Lys28, Lys192, Lys491, Lys492, Lys509, Lys520, and Lys521 (Figure 7 and Table 2). Namely, ubiquitination of Lys28 and Lys192 were identified through high-throughput proteomic analyses [215,216], but the biological significance of the ubiquitination of those Lys residues in MUTYH is unknown. On the contrary, Dorn et al. (2014) demonstrated that the E3 ubiquitin ligase Mule/HUWE1 interacts with and ubiquitinates MUTYH’s C-terminal region (amino acids 475–535), resulting in downregulation of MUTYH [217]. Within that region, the mutation of five Lys resides (Lys491, Lys492, Lys509, Lys520, and Lys521) to Arg resulted in augmented MUTYH in HEK293T cells. Ubiquitination regulates MUTYH’s cellular localization and repair capacity during oxidative stress. Cellular fractionation and immunofluorescence assays showed that ubiquitinated MUTYH is retained mainly in the cytoplasm, while ubiquitin-free localizes to nuclei and associates with chromatin.

DNA damage is a common feature of Acute Kidney Injury (AKI), induced by ischemia and genotoxic agents such as cisplatin [225]. Recently, Yang and colleagues (2025) showed that patients and mice models of AKI have decreased levels of MUTYH [226]. Furthermore, *MUTYH* knockout mice had higher expression of markers of Cisplatin-induced AKI compared to WT, and overexpression of nuclear MUTYH isoforms ameliorated AKI and Cisplatin-induced DNA damage. Proteomic analysis of cisplatin-treated cells showed upregulation of Mule/HUWE1 ubiquitin ligase and Mule/HUWE1-MUTYH enrichment in cisplatin-treated cells [226]. These results suggest that Mule/HUWE1 tightly controls MUTYH protein turnover.

#### 3.3.3. PARylation

A feature of MUTYH-mediated DNA repair is the rapid activation of PARP-1 upon oxidative DNA damage [19,227]. As mentioned above, accumulation of nuclear OG drives PARP1-dependent nuclear translocation of AIF, DNA fragmentation, and ultimately, apoptosis [19] (Figure 5). PARP1 association with MUTYH was initially proposed as a DNA repair-triggered signaling event. In 2019, Hendriks et al. identified multiple PARylation sites in MUTYH (Ser24, Ser511, Ser505, Ser508, and Ser532) [218] (Figure 7). The functional consequences of this remain unknown. PARylation is known to regulate key aspects of DNA repair, including DNA association [228], recruitment of downstream BER proteins [229], and deubiquitination to prevent degradation [230]. As such, although the role of PARylation of MUTYH is still unclear, it is likely that is represents an additional regulatory role controlling MUTYH-mediated repair.

### 3.4. MUTYH’s Regulation by Protein–Protein Interactions

MUTYH is subjected to a wide network of PPIs linking MUTYH function to cellular process beyond BER, including interactions with other DNA repair pathways (MMR and NER), as well as DNA replication, cell cycle control and differentiation, proteasomal degradation, transcription, and chromatin organization (Figure 5). The effects of some PPI on MUTYH function are detailed in the following sections.

#### 3.4.1. MUTYH-MutSα: Avoidance of Pro-Mutagenic Activity of MUTYH

MUTYH might channel BER toward pro-mutagenic repair if it removes A from the template strand after misincorporation of G across A by DNA polymerases. Such misincorporation might derive from replicative DNA polymerases (α, δ and ε) [231,232,233], or even from DNA repair polymerases (Polβ) [234]. Thus far, it is not known how such a pro-mutagenic scenario is avoided. Although MutY homologs exhibit adenine excision from A:G mismatches in vitro, such activity is negligible in a cellular context [164,165]. This suggests that MutY/MUTYH-mediated repair activity on A:G mismatches might be negatively regulated in cells. A potential regulatory mechanism involves MUTYH and MMR interacting, specifically through the MSH2 and MSH6 proteins that form the MutSα heterodimeric complex of MMR (Figure 7). MutSα recruits downstream MMR factors via MutL homologs [235], and its interaction with MUTYH maps to the [4Fe-4S] cluster motif [219]. At low concentrations of MutSα (0.25 to 4 nM), MUTYH affinity for A:OG-containing DNA duplexes increases up to 8-fold, with a 2-fold enhancement in adenine excision activity [219]. Higher concentrations of MutSα (>6 nM) inhibit MUTYH activity by 15%. Additionally, MutSα binds A:G and A:OG mispairs comparably (58 and 78 nM, respectively) [236], while MutY shows a 50-fold preference for an uncleavable analog of A opposite OG vs. G [237].

These observations support a model in which MutSα negatively regulates MUTYH toward A:G mismatches. At low stoichiometric ratios, interaction with MutSα enhances intrinsic preference for A:OG substrates. However, upon OG-independent replicative stress, where A is mis-incorporated across G, enriched MutSα recruitment and its stoichiometric excess over MUTYH may competitively inhibit MUTYH activity, preserving specificity for OG:A. Genomic OG accumulation in MMR-deficient mouse embryonic fibroblasts [238,239] and the downregulation of MSH2 and MSH6 in cells treated with non-cytotoxic concentration of H_2_O_2_ [240] might favor MUTYH-mediated OG:A repair.

#### 3.4.2. PCNA and 9-1-1 Complex as Structural and Functional Scaffolds for MUTYH-Associated BERosome Assembly

BER has been proposed to proceed through the assembly of a macromolecular repair complex, dubbed the “BERosome,” at sites requiring repair [241]. Given the critical nature of MUTYH-derived repair intermediates [16,17,19], the formation of a MUTYH-associated BERosome likely ensures that enzymes required for efficient and faithful OG:A repair are co-localized at the repair site, protecting and processing genotoxic intermediates. This is particularly important because OG is on the opposite strand and the associated repair polymerase Polλ, which preferentially incorporates A over C (*k_cat_*/*K_M_*; 42 vs. 11 min^−1^ μM^−1^, respectively) [242]. Indeed, MUTYH and APE1 enhance Polλ fidelity in vitro by biasing towards C incorporation [243], Thus, the formation of a BERosome comprised of all these enzymes could function as a proofreading hub, allowing rapid re-initiation of repair by MUTYH if adenine is mis-incorporated.

PCNA is a homotrimer, ring-shaped DNA sliding clamp that encircles DNA and plays a central role in DNA replication and repair [244]. PCNA’s PPI are mediated by its interdomain connector loop (IDCL) and PCNA-interacting protein (PIP) box. The Lu-Chang Lab identified a C-terminal PIP box (QXX(I/L/M)XXFF) in MUTYH [245] (Figure 7), establishing MUTYH as a PCNA-interacting protein, supporting its role in replication-associated repair [220]. Indeed, the disruption of the PCNA-MUTYH binding interface abrogates OG:A repair in vivo [246]. PCNA interacts with downstream proteins like MUTYH [247], APE1 [248], and Polλ [249]. Likewise, MUTYH interacts with APE1 [250] and Polλ [250]. During oxidative stress, MUTYH is recruited to damaged DNA along with PCNA and Polλ [249]. Since PCNA contains three PPI IDCL motifs, it can bind to three different protein partners, suggesting a scaffolding role for a MUTYH-associated BERosome during S phase.

The Rad9-Rad1-Hus1 (9-1-1) complex is another sliding clamp which also has a donut-like structure composed of the Rad9, Rad1, and Hus1 subunits [251]. The 9-1-1 complex is loaded onto the lesion site, activating the cell cycle checkpoint and distinct DNA repair pathways [252]. It physically interacts with and modulates the activity of MUTYH [221,247] and other BER enzymes [137,253,254,255,256,257,258]. MUTYH physically interacts with the 9-1-1 complex through its Rad9 and Hus1 subunits [221,247]. These interactions stimulate MUTYH activity and are enriched upon ionizing radiation and oxidative stress [160,221]. Interestingly, it is reported that MUTYH, APE1, and Hus1 form a stable complex in vitro and in cells following oxidative stress [250]. This suggests that the 9-1-1 complex might assist with and regulate the assembly of the MUTYH-associated BERosome at the cell cycle checkpoint, as is the proposed function of PCNA during DNA replication. However, there is no direct evidence of the formation of the BERosome for OG:A repair. It is possible that multi-BER enzyme complexes may be transient and therefore hard to isolate, but their existence could explain the exquisite regulation of BER and substrate-product hand-off.

#### 3.4.3. Regulation of MUTYH Turnover

MUTYH, like other DGs, including OGG1, has the biochemical feature of limited turnover in vitro. For instance MUTYH has at least 3200-fold higher affinity for its product (AP-site analog [THF]:OG; *K_D_* < 10 pM) than its substrate (A analog [fluorinated-A]:OG; *K_D_* = 32 nM), resulting in a markedly slow turnover (*k*_3_ of 0.02 min^−1^) [167]. This could be explained by MUTYH embracing the AP site:OG-containing DNA after base excision. In a cellular context, this could be a regulatory step, with turnover being mediated through PTMs or PPIs. Protection of AP:OG sites would be vital, given that resulting ssDNA and dsDNA breaks are more cytotoxic than AP:OG [259].

MUTYH activity is impacted by its protein partners. For instance, human APE1 stimulates mouse MUTYH activity on OG:A-containing DNA 60-fold when APE1 is in excess (80:1 APE1:MUTYH ratio). Other proteins have shown similar effects on MUTYH, such as the 9-1-1 complex [260] and SIRT6 [223]. However, there is no direct kinetic evidence for enhancement of MUTYH’s turnover. The increase in activity might result from favoring the formation of enzyme-substrate complexes, as evidenced by the MUTYH-APE1 interaction [222], or increasing the fraction of active protein, where the protein partner might function as a chaperone favoring the proper folding of the inactive protein population [261].

The Van Houten and David laboratories, along with other collaborators, carried out detailed studies to evaluate the impact of NER protein UV-DDB on MUTYH, OGG1, and APE1 [134]. They showed that UV-DDB, with an inherently robust ability to recognize and bind AP-sites, stimulates OGG1, APE1, and MUTYH activities [134]. In fact, UV-DDB stimulates MUTYH, increasing its turnover up to 5-fold [224]. EMSA, atomic force microscopy, and single molecule analysis demonstrated that UV-DDB interacts transiently with MUTYH, inducing its dissociation from OG:AP-site-containing DNA, reducing the half-life of the MUTYH-DNA complex from 8800 to 590 s. Hence, UV-DDB directly regulates MUTYH OG:A repair by favoring the release of its product, the OG:AP site, thus indirectly regulating the subsequent downstream BER reactions. Mutations in the genes encoding UV-DDB or MUTYH that disrupt their interaction, and consequently full repair of OG:A, might contribute in part to the build-up of DNA repair intermediates, compromising cell viability and contributing to MAP etiology.

## 4. Concluding Remarks

Over 30 years of amazing work led by numerous laboratories around the globe have demonstrated that the DNA repair properties of OGG1 and MUTYH are not limited to BER, but also transcriptional regulation and complex signaling events which determine genomic and cellular fate. The interplay these DGs have through their PTMs and PPIs arm OGG1 and MUTYH with the potential to impact distinct cellular aspects beyond BER. Therefore, understanding their regulation at the transcriptional and protein level is pivotal to understanding the functions of these enzymes, as well as their implication in carcinogenesis and disease. Leveraging this knowledge can aid the development of potential drugs to enhance or hijack repair. Finally, there are still questions regarding the mechanisms these enzymes utilize. For example, understanding a general mechanism of product turnover may shed light on the implication of OG repair in telomeric instability and apoptosis. Additionally, understanding BERosome formation could present novel opportunities for therapeutic intervention.

## Data Availability

No new data were created or analyzed in this study.
